# High Percentage of ADAM-10 Positive Melanoma Cells Correlates with Paucity of Tumor-Infiltrating Lymphocytes but Does Not Predict Prognosis in Cutaneous Melanoma Patients

**DOI:** 10.1155/2015/975436

**Published:** 2015-07-22

**Authors:** Piotr Donizy, Marcin Zietek, Marek Leskiewicz, Agnieszka Halon, Rafal Matkowski

**Affiliations:** ^1^Department of Pathomorphology and Oncological Cytology, Wroclaw Medical University, Borowska 213, 50-556 Wroclaw, Poland; ^2^Lower Silesian Oncology Centre, pl. Hirszfelda 12, 53-413 Wroclaw, Poland; ^3^Department of Statistics, Wroclaw University of Economics, Komandorska 118-120, 53-345 Wroclaw, Poland; ^4^Department of Oncology and Division of Surgical Oncology, Wroclaw Medical University, pl. Hirszfelda 12, 53-413 Wroclaw, Poland

## Abstract

ADAM-10 (CDw156, CD156c, and kuzbanian) is a protein belonging to a superfamily of metalloproteases, enzymes capable of degrading the extracellular matrix. ADAMs have also been shown to be primarily involved in ectodomain cleavage. The aim of the study was to assess the expression and intracellular location of ADAM-10 in 104 primary skin melanomas and 16 metastatic lesions from regional lymph nodes. Also, prognostic significance of ADAM-10 expression in primary tumor cells and metastatic lesion cells was evaluated during 5-year observation. It was revealed that high expression of ADAM-10 positive cells was strictly related with lower intensity of tumor-infiltrating lymphocytes (*P* = 0.037), which suggests that ADAM-10 regulates immunoresponse in melanoma initiation and progression. No statistically significant correlations were found between ADAM-10 expression in primary tumor cells and nodal metastases and other histopathological parameters analyzed. Decreased immunoreactivity of ADAM-10 in cancer cells from regional lymph nodes was correlated with worse prognosis; however this correlation was statistically nonsignificant (*P* = 0.065). Review of the literature shows that our study is the first one ever to describe the significance of ADAM-10 expression in correlation with detailed histopathological parameters of the primary tumor and data on long-term survival of cutaneous melanoma patients.

## 1. Introduction

ADAM-10 (CDw156, CD156c, and kuzbanian) is a protein belonging to a superfamily of metalloproteases, enzymes capable of degrading the extracellular matrix. ADAMs have also been shown to be primarily involved in ectodomain cleavage [1].

ADAM family proteins are membrane anchored glycoproteins which play a major role in a number of cytobiochemical processes such as proteolysis, intercellular adhesion, and activation of various signaling cascades [2, 3]. The analysis of the molecular structure of ADAMs showed that they exhibit a few characteristic domains which, starting from their N-terminal ends, include a prodomain, a metalloprotease domain, a disintegrin-like domain, EGF-like domain, a cysteine-rich domain, and transmembrane and cytoplasmic fragments [4].

In a normal cell, catalytically active ADAM-10 protein functions as an enzyme shedding extracellular fragments of membrane-bound proteins (shedding), which leads to the release of active protein ectodomains. ADAM-10 also participates in the biochemical process referred to as regulated intramembrane proteolysis (RIP), during which, and with mediation by presenilin-dependent *γ*-secretase, intracellular domain (ICD) fragments are formed, which upon translocation to the nucleus modulate several signal transduction pathways and become either transcriptional activators or repressors [5, 6]. A wide spectrum of protein substrates for ADAM-10 includes molecules that have been shown to be involved in carcinogenesis initiation and cancer progression. They include proteins such as CD44, E-cadherin, Notch, Delta, FasL, and TNF*α* and epidermal growth factor receptors [4].

The aim of the study was to assess the expression and intracellular location of ADAM-10 in 104 primary skin melanomas and 16 metastatic lesions from regional lymph nodes. Also, prognostic significance of ADAM-10 expression in primary tumor cells and metastatic lesion cells was evaluated during 5-year observation. Correlations were also analyzed between ADAM-10 immunoreactivity parameters and detailed clinicopathological parameters such as Breslow thickness and Clark level, presence of nodal and distant metastases, recurrence of primary tumor, mitotic rate, ulceration, lymphocytic inflammatory infiltration, regression, and microsatellitosis.

## 2. Materials and Methods

### 2.1. Patients

The study group consisted of 104 patients with CMM, who were diagnosed between 2005 and 2010 and treated in the Lower Silesian Oncology Center in Wroclaw, Poland. Additionally, tissue material obtained from 16 nodal metastatic foci was included in the study. The group was selected on the basis of tissue material (paraffin blocks and histopathology slides) and the availability of medical documentation. Comprehensive clinical data were obtained from archival medical records. The diagnostic and therapeutic procedures utilized were determined from medical records in the Oncology Outpatient Clinic of the Lower Silesian Oncology Center and data provided by the Lower Silesian Cancer Registry and Civil Register Office. The retrospective study was approved by the Ethical Committee of the Wroclaw Medical University, Poland.

Patients enrolled in the study were treated by then-available methods. Excisional biopsy of the primary lesion was performed. Once cutaneous melanoma was diagnosed in histopathological examination, the primary procedure was extended by excising the scar with a margin of 5, 10, or 20 mm of unaffected skin depending on Breslow thickness and primary tumor location, if any. If nodal metastases (cN0) were not clinically manifested and Breslow thickness was above 1 mm (>pT1a), sentinel lymph node biopsy (SNLB) was performed. When metastases were observed in the regional lymph nodes (found either clinically or by SLNB), lymphadenectomy was performed.

Clinicopathological profile of patients included the following parameters: age and gender, primary tumor location, tumor stratification according to AJCC (pT), presence or absence of nodal (pN) and distant (pM) metastases, information on disease recurrence, and sentinel lymph node biopsy (SLNB) procedures (Table 1).

### 2.2. Tumor Samples and Histopathological Evaluation

Tumor specimens were fixed in 10% buffered formalin and embedded in paraffin. All haematoxylin and eosin stained sections were examined by two pathologists. The parameters of the primary tumor recorded in pathology reports were Breslow thickness, Clark level, growth phase, histologic type, mitotic rate (number of mitotic figures per 1 mm^2^), presence of ulceration, lymphangio invasion, microsatellitosis, intensity of lymphocytic inflammatory infiltrate (TILs: tumor-infiltrating lymphocytes), and microscopic evidence of regression (Table 2).

### 2.3. Immunohistochemistry

Formalin-fixed, paraffin embedded tissue was freshly cut (4 *μ*m). The sections were mounted on superfrost slides (Menzel Glaser, Germany), dewaxed with xylene, and gradually hydrated. The activity of endogenous peroxidase was blocked by 5-minute exposure to 3% H_2_O_2_. The sections were boiled for 15 minutes at 250 W in Antigen Retrieval Solution (DakoCytomation, Denmark). Immunohistochemical reactions were then performed using polyclonal antibody detecting ADAM-10 (clone LS-B324, LifeSpan Biosciences, USA), N-cadherin (clone D-4, Santa Cruz Biotechnology, USA), and SPARC (clone PP16, Santa Cruz Biotechnology, USA). The tested sections were incubated with antibodies for 1 hour at room temperature. The subsequent incubations involved biotinylated antibodies (15 minutes at room temperature) and streptavidin-biotinylated peroxidase complex (15 minutes at room temperature) (LSAB+, HRP, DakoCytomation, Denmark). DAB (Vector Laboratories, UK) was used as a chromogen (10 minutes at room temperature). All sections were counterstained with Meyer's hematoxylin. In every case control reactions were performed, with the relevant antibody substituted by Primary Mouse Negative Control (DakoCytomation, Denmark).

### 2.4. Evaluation of Reaction Intensity

The intensity of the immunohistochemical reaction was estimated independently by two pathologists. Doubtful cases were reevaluated under a double-headed microscope and staining was discussed until consensus was achieved.

ADAM-10 expression was observed only in melanoma cells in both tissue material obtained from the primary tumor and nodal metastatic foci. No ALCAM immunoreactivity was identified in stromal compartment of tumor or lymphocytes from regional lymph nodes. Cancer cells of the primary tumors and metastatic cells displayed predominantly diffuse cytoplasmic expression.

The expression of ADAM-10 protein was calculated using a semiquantitative method. Two immunohistochemical reaction parameters were considered when evaluating the expression of the foregoing proteins: the percentage of cells with a positive cytoplasmic reaction (the percentage of reactive tissue) and the reaction intensity. The final immunohistochemical reaction results are expressed according to the semiquantitative IRS (ImmunoReactive Score) scale devised by Remmele and Stegner [7]. This scale assigns a score for the percentage of cells demonstrating reaction (0–4 points) and for reaction intensity (0–3 points). The final result is the product of the scores for these two parameters (0–12 points) and is referred to as an IRS factor or score.

### 2.5. Statistical Analysis

Statistical analysis was performed using the Statistica 10.0 and IBM SPSS 21 software packages. Overall survival (OS) was defined as the time between the primary surgical treatment and death, and OS was censored at last follow-up for patients who were still alive. A *χ* ^2^ test, exact Fisher test in the case of 2 × 2 tables, and Spearman rank correlation were used to analyze associations between mitotic rate and the presence of ulceration and clinicopathological parameters. Differences between the means were tested with a nonparametric test (Mann-Whitney *U* test and Kruskal-Wallis test); the log-rank test was used to compare survival in two groups, the overall survival rate was estimated by the Kaplan-Meier method, and the influence of explanatory variables on death risk was analyzed by means of the Cox proportional hazard regression. *P* values < 0.05 were considered statistically significant.

## 3. Results

### 3.1. ADAM-10 Expression in Primary Tumors and Nodal Metastases

**Figure 1:**
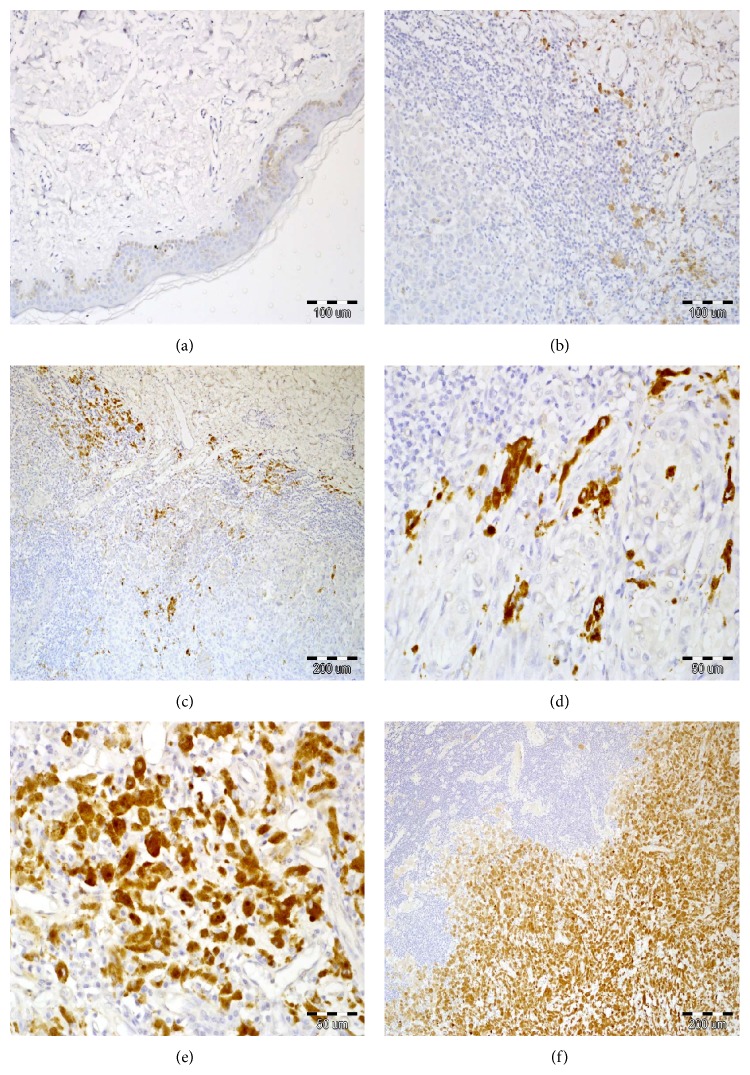
Immunohistochemically visualized expression of ADAM-10 in cutaneous melanoma. Lack of ADAM-10 immunoreactivity in normal skin ((a), 200x, hematoxylin). Low percentage of ADAM-10-positve cells in cutaneous melanoma with brisk tumor-infiltrating lymphocytes ((b), 10% of ADAM-10 positive cells, 200x; (c), hematoxylin, 30% of ADAM-10 positive cells, 200x, hematoxylin). Intermediate percentage of ADAM-10 positive cells in cutaneous melanoma with nonbrisk tumor-infiltrating lymphocytes ((d), 40% of ADAM-10 positive cells, 400x). Enhanced ADAM-10 immunoreactivity in melanoma cells with scanty tumor-infiltrating lymphocytes ((e), IRS 12, 400x, hematoxylin). High cytoplasmic reactivity of ADAM-10 in regional lymph node metastases ((f), 200x, hematoxylin).

ADAM-10 expression defined as IRS > 0 was found in 82 primary tumors (78.8% of patients (Figure 1)). No ADAM-10 immunoexpression was revealed in 22 primary tumors (21.2%). Average IRS was 4.08 ± 3.04. In nodal metastases ADAM-10 reactivity was shown in 9 out of 16 cases (56.2%), and no expression (IRS = 0) was found in 7 patients (43.8%). Average IRS for nodal metastases was 3.50 ± 4.31.

### 3.2. Correlations between ADAM-10 Expression Parameters in Primary Tumor and Metastases with Histopathological Features of Primary Melanoma

It was revealed that high expression of ADAM-10 positive cells was strictly related with lower intensity of tumor-infiltrating lymphocytes (*P* = 0.037), which suggests that ADAM-10 regulates immunoresponse in melanoma initiation and progression. No statistically significant correlations were found between ADAM-10 expression in primary tumor cells and nodal metastases and other histopathological parameters analyzed (Table 3).

### 3.3. Correlations between ADAM-10 Immunoreactivity and Clinicopathological Parameters

It was revealed that ADAM-10 expression in primary tumor was reduced with increasing age at diagnosis (*P* = 0.029). Interestingly, lower ADAM-10 expression in cancer cells from nodal metastases was also correlated with older age at diagnosis (*P* = 0.037) (Table 3).

Statistical analysis showed statistically nonsignificant trend of increased ADAM-10 immunoexpression in cancer cells from primary tumor and the presence of distant metastases (*P* = 0.088). Other clinical parameters such as pT, pN, recurrence, sex, and location of the primary tumor did not show any significant correlations with ADAM-10 expression (Table 3).

### 3.4. Analysis of ADAM-10 Expression Effect on 5-Year Survival in Melanoma Patients

Decreased immunoreactivity of ADAM-10 in cancer cells from regional lymph nodes was correlated with worse prognosis; however this correlation was statistically nonsignificant (*P* = 0.065). Other parameters of ADAM-10 expression in primary tumor and nodal metastasis cells did not have a statistically significant impact on the prognosis in cutaneous melanoma patients in the analyzed group of patients.

### 3.5. Analysis of the Correlations between ADAM-10 Expression Parameters and SPARC and N-Cadherin Immunoreactivity in Melanoma Cells

Statistical analysis revealed a significant correlation between enhanced immunoreactivity of ADAM-10 and augmented expression of N-cadherin and SPARC in primary tumor cells and melanoma cells in nodal metastases (Table 4). N-Cadherin and SPARC are crucial proteins for the induction and progression of EMT (epithelial-mesenchymal transition) and these results may be associated with the potential role of ADAM-10 in the process of EMT.

## 4. Discussion

A review of the literature (PubMed, 1970–2015; keywords: ADAM-10 and melanoma) revealed only two articles that describe immunohistochemical analysis of ADAM-10 expression in cutaneous melanoma [8, 9].

Lee et al. [8] analyzed ADAM-10 immunoexpression based on 46 primary cutaneous melanomas and 127 melanoma metastases (106 carcinomas were distant organ metastases, and only 21 cases were nodal metastases). It must be stressed that the researchers did not assess ADAM-10 expression in the whole tumor but only in a section of tumor tissue using tissue microarrays (TMA) [8]. Lee et al. [8] observed no ADAM-10 expression in 35% of primary tumors (16/46), whereas in our study the percentage was 21.2% (22/104). Lee et al. [8] also showed no ADAM-10 expression in metastases in 26% of cases (33/127). In our study the percentage was 43.8% (7/16). It must be stressed that the data is not comparable or compatible since, in the metastatic carcinoma group studied by Lee et al. [8], distant organ metastases were the dominant ones (106/127), while nodal metastases constituted only a small percentage (21/127; 16%). Our study was entirely focused on ADAM10 expression in cancer metastases from regional lymph nodes.

Similarly to our results, Lee et al. [8] did not show statistically significant correlations between Breslow thickness and ADAM-10 overexpression in cancer cells.

Lee et al. [8] showed that ADAM-10 immunoreactivity was significantly higher in metastases than in primary tumor (*P* = 0.04), which is not compatible with our results. Interestingly, our statistical analysis showed statistically nonsignificant trend of increased ADAM-10 immunoexpression in cancer cells from the primary tumor and the presence of distant metastasis (*P* = 0.088). Other clinical parameters such as pT, pN, sex, or location of the primary tumor were not shown to be significantly correlated with ADAM-10 expression.

It must be stressed that in our study it was only ADAM-10 immunoreactivity in metastases that had prognostic value. Analysis of Kaplan-Meier curves showed that decreased expression of ADAM-10 in cancer cells from regional lymph node metastases was correlated with worse prognosis; however this correlation was statistically nonsignificant (*P* = 0.065).

Anderegg et al. [9] analyzed ADAM-10 expression in melanoma cells only as regards its enzymatic activity related with CD44. 16 cases of melanoma and cell lines were analyzed in the paper, which confirmed that ADAM-10 was critically involved in enzymatic treatment of CD44 [9]. ADAM-10 influence on long-term survival or the correlation of ADAM-10 expression with histopathological parameters of the primary tumor was not studied. Moreover, we also revealed a significant correlation between enhanced immunoreactivity of ADAM-10 and augmented expression of N-cadherin and SPARC in primary tumor cells and melanoma cells in nodal metastases which may be associated with the potential role of ADAM-10 in the process of EMT [10, 11]. It must be stressed that it is a hypothesis that needs to be supplemented and validated empirically with a much larger study group and animal models of metastasis; however, it is a voice in the discussion and confirms the hypotheses concerning the role of ADAM-10 in melanoma progression.

An innovative feature of our study was the finding that decreased intensity of tumor-infiltrating lymphocytes (TILs) was strictly correlated with the increased number of ADAM-10 positive cancer cells in the primary tumor (*P* = 0.037). The possible meaning of this correlation is unclear and needs further studies, yet it suggests that ADAM-10 is involved in the regulation of immunological response in the initiation and progression of melanoma. Neither of the research teams described in the quoted papers analyzed or correlated ADAM-10 expression parameters with the details of the histopathology report [8, 9]. Furthermore, it was shown that ADAM-10 expression in the primary tumor (*P* = 0.029) was reduced with increasing age at diagnosis and that decreased expression of ADAM-10 in cancer cells from nodal metastases was also correlated with more advanced age at diagnosis (*P* = 0.037). Lee et al. [8] and Anderegg et al. [9] did not study the potential impact of age on ADAM-10 expression in cancer cells.

To sum up, it must be pointed out that, in the two papers referred to above by Lee et al. [8] and Anderegg et al. [9], only the expression patterns of ADAM-10 were studied and the impact on long-term survival was not analyzed. Review of the literature shows that our study is the first one ever to describe the significance of ADAM-10 expression in correlation with detailed histopathological parameters of the primary tumor and data on long-term survival of cutaneous melanoma patients.

## Figures and Tables

**Table 1:** Clinicopathological characteristics of the patients.

Clinicopathological characteristics	Number (%)
All patients	104 (100.0)

Age in years (21–79)	
Mean: 56.5 ± 15.4; median: 58.5	
Gender	
Female	60 (57.7)
Male	44 (42.3)
Primary tumor location	
Head/neck	15 (14.4)
Upper extremity	18 (17.3)
Lower extremity	25 (24.0)
Trunk	42 (40.4)
Hand/foot	4 (3.8)
Primary tumor (pT)	
pT1	34 (32.7)
pT2	20 (19.2)
pT3	27 (26.0)
pT4	23 (22.1)
Sentinel lymph node biopsy status (SNLB)	60 (57.7)
No metastases (SNLB−)	48 (80.0)
Metastases present (SNLB+)	12 (20.0)
Regional lymph nodes status (pN)	
No metastases (pN−)	86 (82.7)
Metastases present (pN+)	18 (17.3)
Recurrence	
No	87 (83.7)
Yes	17 (16.3)
Distant metastases	
No	99 (95.2)
Yes	5 (4.8)

**Table 2:** Histopathological parameters of primary tumors.

Histopathological parameters of the primary tumor	Number (%)
Breslow thickness	
	34 (32.7)
1.01–2.00 mm	20 (19.2)
2.01–4.00 mm	27 (26.0)
	23 (22.1)
Clark level	
I	0 (0.0)
II	18 (17.3)
III	49 (47.1)
IV	26 (25.0)
V	11 (10.6)
Histologic type	
Superficial spreading melanoma (SSM)	68 (65.4)
Nodular malignant melanoma (NMM)	32 (30.8)
Acral-lentiginous melanoma (ALM)	4 (3.8)
Mitotic rate	
0	45 (43.3)
1-2	26 (25.0)
≥3	33 (31.7)
Ulceration	
No	55 (52.9)
Yes	49 (47.1)
Lymphangio invasion	
No	74 (71.2)
Yes	30 (28.8)
Growth phase	
Radial	3 (2.9)
Vertical	101 (97.1)
Tumor-infiltrating lymphocytes (TILs)	
No	18 (17.3)
Nonbrisk	34 (32.7)
Brisk	52 (50)
Microsatellitosis	
No	98 (94.2)
Yes	6 (5.8)
Tumor regression	
No	96 (92.3)
Yes	8 (7.7)

**Table 3:** Correlations between clinicopathological and histopathological characteristics and ADAM-10 expression parameters in primary tumors and nodal metastases.

	ADAM-10 expression, primary tumor			ADAM-10 expression, nodal metastasis		
Clinicopathological parameters	%	Int	IRS	%	Int	IRS

pT^a^	0.965	0.292	0.437	0.528	0.424	0.424
pN^b^	0.195	0.757	0.820	0.262	0.273	0.477
Distant metastases^b^	0.292	***0.088***	0.204	0.482	0.673	0.934
Recurrence^b^	0.633	0.952	0.989	0.390	0.092	0.243
Age^a^	0.785	0.145	**0.029**	**0.043**	**0.037**	**0.037**
Gender^b^	0.305	0.953	0.952	0.696	0.574	0.740
Primary tumor location^c^	0.213	0.180	0.424	0.825	0.546	0.447

Clinicopathological parameters	%	Int	IRS	%	Int	IRS

Breslow thickness^a^	0.640	0.265	0.363	0.558	0.420	0.394
Clark level^a^	0.229	0.945	0.899	0.785	0.466	0.909
Growth phase^b^	0.614	0.264	0.220	100% vertical growth phase		
Histologic type^c^	0.462	0.299	0.160	0.390	0.500	0.342
Mitotic rate^a^	0.319	0.367	0.552	0.845	0.140	0.248
Ulceration^b^	0.367	0.603	0.582	0.720	0.847	0.800
Lymphangio invasion^b^	0.290	0.531	0.485	0.631	0.205	0.212
Microsatellitosis^b^	0.398	0.275	0.551	0.631	0.300	0.307
Tumor-infiltrating lymphocytes (TILs)^c^	**0.037**	0.459	0.775	0.987	0.105	0.256
Tumor regression^b^	0.597	0.073	0.228	100% without regression		

^a^ *P* value of Spearman rank correlation.

^b^ *P* value of Mann-Whitney *U* test.

^c^ *P* value of Kruskal-Wallis test.

Statistically significant results (*P* < 0.05) are in bold font.

**Table 4:** Correlations between ADAM-10 expression parameters in primary tumors and nodal metastases and SPARC and N-cadherin immunoreactivity in melanoma cells in primary tumors and nodal metastases.

Expression of ADAM-10	Expression of SPARC in primary tumor			Expression of SPARC in nodal metastasis			Expression of N-cadherin in primary tumor			Expression of N-cadherin in nodal metastasis		
	%^a^	Int^b^	IRS^c^	%	Int	IRS	%	Int	IRS	%	Int	IRS
Primary tumor												
%	**0.006**	0.197	**0.027**	0.190	0.128	0.135	0.120	0.364	0.109	0.095	0.335	0.198
Int	**<0.001**	**<0.001**	**<0.001**	0.100	0.101	0.064	**<0.001**	**<0.001**	**<0.001**	**0.029**	**0.015**	**0.015**
IRS	**<0.001**	**<0.001**	**<0.001**	**0.010**	**0.041**	**0.007**	**<0.001**	**<0.001**	**<0.001**	**<0.001**	**0.003**	**<0.001**

Nodal metastasis												
%	0.193	0.149	0.128	**0.001**	0.066	**0.004**	0.393	0.060	0.212	**0.004**	**0.024**	**0.004**
Int	0.107	0.279	0.124	**<0.001**	**<0.001**	**<0.001**	0.288	0.052	0.110	**0.022**	**0.001**	**0.002**
IRS	0.080	0.084	**0.041**	**<0.001**	**<0.001**	**<0.001**	0.325	**0.012**	0.058	**0.001**	**<0.001**	**<0.001**

^a^Percentage of protein-positive melanoma cells (0–4 points).

^b^Intensity of immunohistochemical reaction (0–3 points).

^c^ImmunoReactive Score (product of the scores for the percentage of positive cells and intensity of reaction (0–12 points)).

*P* value of Spearman rank correlation; statistically significant results (*P* < 0.05) are in bold font.

## References

[1] Mochizuki S., Okada Y. (2007). ADAMs in cancer cell proliferation and progression. *Cancer Science*.

[2] Edwards D. R., Handsley M. M., Pennington C. J. (2008). The ADAM metalloproteinases. *Molecular Aspects of Medicine*.

[3] Blobel C. P. (2005). ADAMs: key components in egfr signalling and development. *Nature Reviews Molecular Cell Biology*.

[4] Crawford H. C., Dempsey P. J., Brown G., Adam L., Moss M. L. (2009). ADAM10 as a therapeutic target for cancer and inflammation. *Current Pharmaceutical Design*.

[5] Seals D. F., Courtneidge S. A. (2003). The ADAMs family of metalloproteases: multidomain proteins with multiple functions. *Genes and Development*.

[6] Wolfe M. S., Kopan R. (2004). Intramembrane proteolysis: theme and variations. *Science*.

[7] Remmele W., Stegner H. E. (1987). Recommendation for uniform definition of an immunoreactive score (IRS) for immunohistochemical estrogen receptor detection (ER-ICA) in breast cancer tissue. *Pathologe*.

[8] Lee S. B., Schramme A., Doberstein K. (2010). ADAM10 is upregulated in melanoma metastasis compared with primary melanoma. *Journal of Investigative Dermatology*.

[9] Anderegg U., Eichenberg T., Parthaune T. (2009). ADAM10 is the constitutive functional sheddase of CD44 in human melanoma cells. *Journal of Investigative Dermatology*.

[10] Musumeci G., Coleman R., Imbesi R. (2014). ADAM-10 could mediate cleavage of N-cadherin promoting apoptosis in human atherosclerotic lesions leading to vulnerable plaque: a morphological and immunohistochemical study. *Acta Histochemica*.

[11] Paudel S., Kim Y.-H., Huh M.-I. (2013). ADAM10 mediates N-cadherin ectodomain shedding during retinal ganglion cell differentiation in primary cultured retinal cells from the developing chick retina. *Journal of Cellular Biochemistry*.

